# Aerobic physical training impact on adipokines in women with polycystic ovary syndrome – Effects of body fat percentage

**DOI:** 10.20945/2359-3997000000503

**Published:** 2022-08-04

**Authors:** Hugo Celso Dutra de Souza, Stella Vieira Philbois, Tábata de Paula Facioli, Rui Alberto Ferriani, Ada Clarice Gastaldi

**Affiliations:** 1 Universidade de São Paulo Faculdade de Medicina de Ribeirão Preto Departamento de Ciências da Saúde Ribeirão Preto SP Brasil Departamento de Ciências da Saúde, Faculdade de Medicina de Ribeirão Preto, Universidade de São Paulo, Ribeirão Preto, SP, Brasil; 2 Universidade de São Paulo Faculdade de Medicina de Ribeirão Preto Departamento de Ginecologia e Obstetrícia Ribeirão Preto SP Brasil Departamento de Ginecologia e Obstetrícia, Faculdade de Medicina de Ribeirão Preto, Universidade de São Paulo, Ribeirão Preto, SP, Brasil

**Keywords:** Polycystic ovary syndrome, adipokines, aerobic physical training, inflammatory markers, body fat

## Abstract

**Objective:**

We investigated the effects of aerobic training on adipokine concentrations in women with polycystic ovary syndrome (PCOS).

**Subjects and methods:**

120 women, including 60 with PCOS and 60 without PCOS, were divided into six groups (n = 20) based on body fat percentages of 22%-27%, 28%-32%, and 33%-37%. All groups were submitted the same evaluations before and after 16 weeks of aerobic training. These included anthropometric and hemodynamic analyses, cardiopulmonary tests, and laboratory tests. Two-way analysis of variance was performed to evaluate the differences between women with and without PCOS, effect of the body fat percentage, and effect of aerobic training.

**Results:**

Body fat and PCOS were associated with high values of blood glucose, insulin, and testosterone. Body fat also reduced adiponectin levels and increased leptin, tumor necrosis factor-alpha (TNF-α), and interleukin-6 (IL-6). In contrast, the PCOS increased only TNF-α and IL-6 levels. In the PCOS group, aerobic training reduced insulin, triglycerides, leptin, and IL-6 levels. It also promoted an increase in adiponectin and high-density lipoprotein levels. However, aerobic training did not alter TNF-α concentrations.

**Conclusion:**

The body fat potentiates metabolic impairments that may be harmful to women with PCOS. Aerobic training appears to promote an important beneficial effect on the metabolic regulation of adipokines, except TNF-α.

## INTRODUCTION

Polycystic ovary syndrome (PCOS) affects a large proportion of women of reproductive age ( 1 , 2 ). This high prevalence is worrying, as these women commonly have increased body fat percentages and insulin resistance. They also experience changes in the concentration of adipokines secreted by the adipose tissue. This leads to high risks of comorbidities, such as type 2 diabetes and cardiovascular diseases (CVDs) ( 3 – 5 ).

Adipokines are associated with several metabolic activities, such as glycemia regulation, insulin sensitivity, endothelial homeostasis, fat metabolism, immunity, and inflammatory responses ( 6 – 9 ). In women with PCOS, it is common to observe increased levels of tumor necrosis factor alpha (TNF-α), and interleukin 6 (IL-6). This is in addition to reduced adiponectin levels compared to women without PCOS ( 4 , 5 , 10 – 12 ).

Aerobic exercise presents significant beneficial results in the regulation of insulin sensitivity, glucose metabolism, and lipid profiles for various conditions ( 13 – 16 ), including PCOS. Aerobic exercise is valuable and has been widely used for the treatment of these and other disorders ( 17 , 18 ). However, little is known about its effects on serum adipokine concentrations ( 19 ). Thus, the aim of this study was to investigate the effect of aerobic physical training on hemodynamics and the serum concentrations of leptin, adiponectin, TNF-α, and IL-6 in women with PCOS. The women were stratified based on the body fat percentage, as this parameter represents an important variable that affects adipokine concentrations.

## SUBJECTS AND METHODS

### Participants

The current study was a clinical trial that initially included 185 volunteers (18-39 years of age), all the volunteers, with and without PCOS were screened at the Gynecology and Obstetrics Clinic of the Ribeirão Preto Medical School's Hospital (HCFMRP/USP). The volunteers without PCOS were routine outpatient care patients, and all underwent the same procedures and examinations as the volunteers with PCOS, example the regular menstrual cycle and confirmation of the normal androgen levels. Patients with PCOS were diagnosed based on the Rotterdam consensus ( 20 ). The volunteers were divided into six experimental groups (n = 20) based on body fat percentages (22%-27%; 28%-32%; 33%-37%) ( 21 ). The division of these groups aimed to differentiate the effects of PCOS from those resulting from the percentage of body fat. The inclusion criteria were comprised of a negative history of the following: a) smoking, b) cognitive disturbances, c) pregnancy, d) musculoskeletal disorders, e) CVDs and f) use of any medication, including contraceptives for the last 6 months. The study was approved by the Ethics Committee of the Ribeirão Preto Medical School's Hospital (CAAE: 0459.0.004.000-08). The scientific and legal aspects were disclosed to the volunteers. The participants signed a free and informed consent form agreeing to participate. The authors confirm that all ongoing and related trials for the study were registered in the Brazilian Clinical Trials Registry (RBR-4qsf57).

### PCOS diagnosis

Transvaginal pelvic ultrasound was performed using a Voluson 730 Expert Machine (GE Medical Systems, ZIPF, Austria) to analyze the presence or absence of cysts. The ovarian volume and follicle number/size were evaluated. The ovarian volume of the prolate ellipsoid was calculated using the formula depth × width × length × 0.5 ( 22 ).

In addition, the exclusion criteria for PCOS diagnosis were based on the results of laboratory tests for total serum testosterone, androstenedione, sex hormone-binding globulin, free androgen, prolactin, 17-hydroxyprogesterone, and thyrotropin levels. Blood samples were collected during the follicular phase in women with regular ovulatory cycles and at any time for those with irregular cycles. All the above laboratory tests for diagnosis were collected at the HCFMRP/USP laboratory between 07:00 and 10:00 a.m. following a 12-hour fast.

### Protocols

Data were collected in the morning during two laboratory visits, between 07:00 and 10:00 a.m., with a 48-hour interval. Data were collected during the follicular phase for all women with regular ovulatory cycles and at any time for those with irregular cycles. The first assessment included blood collection at the HCFMRP/USP laboratory.

The second visit was performed at the Laboratory of Exercise Physiology and Cardiovascular Physiotherapy of Ribeirão Preto Medical School. During this visit, the following exams were completed: anthropometric measurements and cardiopulmonary function tests. The duration of each visit was approximately 1 hour.

All volunteers were asked to avoid drinking alcoholic beverages and exercise for 48 hours before the assessments. They were also asked to maintain their usual diet for 48 hours before the assessments. All participants were advised to sleep for at least 7-8 hours the night before the visits.

#### Laboratory tests

All volunteers were asked to fast for 12 hours before the assessments. Blood samples (3.5 mL, BD Vaccutainer^®^ EDTA – Becton, Dickin, and Company, Franklin Lakes, NJ, USA) were used to analyze fasting glycemia (hexoquinase-UV) and insulin (chemiluminescence immunoassay), triglyceride (desidrogenase), and total cholesterol and fraction (esterase-oxidase) levels. For adiponectin and leptin analysis, a radioimmunoassay method was used, while the analyses of IL-6 and TNF-α were performed using chemiluminescent immunoassay. Insulin resistance was assessed using the homeostasis model assessment-insulin resistance (HOMA-IR) index ( 23 ).

#### Anthropometric parameters

Body weight and height were obtained using an analog scale with an altimeter (Welmy, Santa Bárbara d’Oeste, São Paulo, Brazil). The body mass index (BMI) was calculated using the formula W/H^2^, where W is the weight in kilograms and H is the height of the subject in meters. Body composition was evaluated using the bioelectrical impedance method (Quantum BIA 101; Q-RJL Systems, Clinton Township, Michigan, USA).

#### Hemodynamic parameters

Heart rate (HR) data were obtained using an electrocardiographic digital recorder (ML866 PowerLab, ADInstruments, Bella Vista, Australia). The blood pressure (BP) was monitored using the auscultatory method using a stethoscope and sphygmomanometer.

#### Cardiopulmonary test

An incremental treadmill exercise test (Super ATL Millenium^®^, Inbramed/Inbrasport, Porto Alegre, RS, Brazil) was performed. A submaximal test was established with the sum of the baseline HR and 85% of the reserve HR (maximum HR – basal HR), following the previously described modified Bruce protocol. Electrical activity was monitored using an electrocardiogram with one lead (CM5). Oxygen and dioxide carbon uptake (VO_2_ and VCO_2_, respectively) were obtained using a metabolic analyser (Ultima™ CardiO2, Medical Graphics Corp., St. Paul, Minneapolis, USA).

### Aerobic physical training

Aerobic training sessions were conducted at the Laboratory of Exercise Physiology and Cardiovascular Physiotherapy of the Ribeirão Preto Medical School. The sessions were performed in groups. They were supervised, monitored, and performed three times per week for 16 weeks on a motorized treadmill. The training intensity was calculated as the sum of HR at rest and 70 %-80% of reserve HR, obtained by means of the following equation: HR recorded at the peak of cardiopulmonary testing – HR at rest. The duration of the training sessions was 1 hour. They were divided into three phases as follows: 5-minutes of warm-up using an intensity lower than the target HR training range (50%-65% of reserve HR), 50-minutes of training using the training HR (70%-80% of reserve HR), and 5-minutes of cool-down using an intensity lower than the training HR (40%-50% of reserve HR). There was an adaptation period during the first 2 weeks of the study during which participants went through 20-30-minute sessions for familiarization and adaptation to the training protocol. The intensity used was equivalent to the sum of HR at rest and 50%-60% of the reserve HR, followed by increases in intensity and duration in the subsequent weeks until the volunteers reached the training HR as described. The participants HRs were monitored throughout the sessions using a pulse frequency meter (Polar RS810).

Regarding adherence, monitoring, and motivation strategies, an individualized training form was completed every day. The supervisor noted the training date and measured the HR, BP, and subjective perception of effort of the volunteers before, during, and after the training session using the Borg scale. Only volunteers who had 90% adherence were included in the final analysis. During all training sessions, the supervisor spoke with the volunteers regarding the importance of regular physical exercise and provided information concerning PCOS. In addition, questions that arose during the training were answered.

### Statistics analysis

All statistical tests were performed with Sigma-Plot^®^, version 11.0 software (Systat Software Inc., San Jose, CA, USA). The Shapiro-Wilk normality test was used, and the data were expressed as the mean ± standard deviation. Two-way analysis of variance, followed by the Student-Newman-Keuls multiple comparison test, was performed to evaluate the differences between women with and without PCOS (PCOS factor), effect of the body fat percentage (body fat factor), and effect of aerobic physical training (training factor). Differences were considered significant at P < 0.05.

## RESULTS

**Figure 1:**
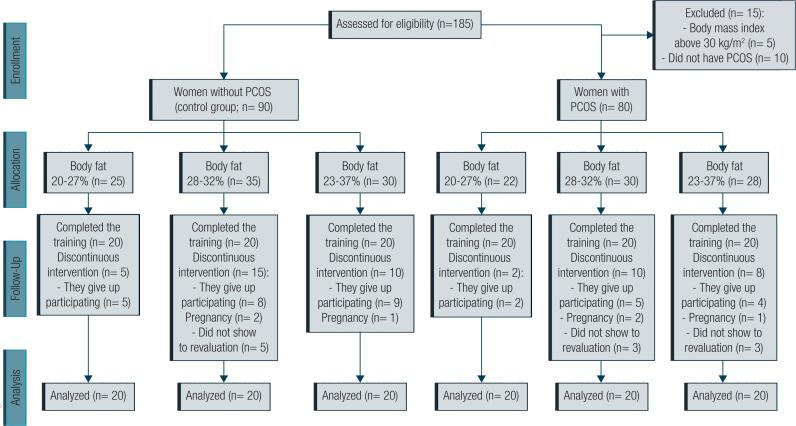
Flow diagram of the study.

In the total of 185 volunteers included in the study only 120 volunteers concluded the training protocol (adherence of > 90%), including 60 with PCOS and 60 without PCOS ( Figure 1 ). Table 1 shows the characteristics and hemodynamic parameters, and Table 2 shows the metabolic parameters and adipokine values of all women with and without PCOS (control groups) distributed according to the body fat percentage. These data were obtained prior to aerobic physical training. Participants belonging to all groups were of similar ages and heights. However, there were differences in weights and BMIs due to body fat percentage distribution. The groups with high body fat percentage showed minor values of VO_2peak_ and major values of high low-density lipoprotein (LDL), triglyceride, and glucose levels. The PCOS groups, independently of body fat percentage, showed higher values in androstenedione levels than control groups, while both body fat and PCOS were associated with higher insulin and testosterone levels and HOMA-IR values. The increase in body fat percentage was associated with low adiponectin levels and high leptin, TNF-α, and IL-6 levels. In turn, PCOS was associated with high TNF-α and IL-6 levels.

**Table 1:** Characteristics and hemodynamic parameters obtained before aerobic physical training in all groups

		22%-27%		28%-32%		33%-37%		PCOS Factor	Body Fat Factor	Interaction
		Control (n = 20)	PCOS (n = 20)	Control (n = 20)	PCOS (n = 20)	Control (n = 20)	PCOS (n = 20)	P	P	P
Characteristics										
	Age, years	32 ± 7	34 ± 5	33 ± 7	35 ± 7	31 ± 4	33 ± 7	0.084	0.686	0.994
	Height, cm	165 ± 7	162 ± 7	166 ± 7	165 ± 5	163 ± 5	161 ± 6	0.109	0.064	0.875
	Weight, kg	63 ± 6	59 ± 4	72 ± 7	69 ± 6	79 ± 11	76 ± 9	0.054	<0.001	0.985
	BMI, kg/m^2^	23.1 ± 2.6	22.6 ± 2.9	26.3 ± 3	25.5 ± 3	29.8 ± 4.3	29.9 ± 4.5	0.410	<0.001	0.955
	Body fat percentage	23.6 ± 1.7	24.4 ± 2.1	29.8 ± 3.2	30.3 ± 1.5	35.7 ± 3.2	35.2 ± 1.5	0.482	<0.001	0.449
Hemodynamic Parameters										
	HR, bpm	74 ± 13	76 ± 10	73 ± 8	78 ± 5	76 ± 12	77 ± 9	0.137	0.855	0.747
	SBP, mmHg	111 ± 11	115 ± 13	113 ± 11	112 ± 9	117 ± 10	118 ± 8	0.627	0.077	0.454
	DBP, mmHg	71 ± 7	73 ± 13	76 ± 9	71 ± 9	75 ± 9	78 ± 9	0.962	0.119	0.139
	MBP, mmHg	87 ± 9	90 ± 13	91 ± 10	87 ± 9	92 ± 9	94 ± 8	0.852	0.094	0.273
	VO_2peak_, mL/kg/min	31 ± 3	30 ± 3	28 ± 5	29 ± 3	26 ± 5	25 ± 6	0.751	<0.001	0.357

Data are presented as mean ± standard deviation. PCOS: polycystic ovary syndrome; BMI, body mass index; HR: heart rate; bpm: beats per minute; SBP: systolic blood pressure; DBP: diastolic blood pressure; MBP: mean blood pressure; VO_2peak_: oxygen consumption at peak of exercise; mL: milliliter; kg: kilogram; min: minute.

**Table 2:** Metabolic and adipokines parameters obtained before aerobic physical training in all groups

		22%-27%		28%-32%		33%-37%		PCOS Factor	Body Fat Factor	Interaction
		Control (n = 20)	PCOS (n = 20)	Control (n = 20)	PCOS (n = 20)	Control (n = 20)	PCOS (n = 20)	P	P	P
Metabolic Values										
	Total Cholesterol, mg/dL	196 ± 17	200 ± 24	212 ± 45	205 ± 32	209 ± 16	219 ± 32	0.708	0.054	0.466
	HDL, mg/dL	45 ± 8	44 ± 8	42 ± 11	42 ± 5	43 ± 7	41 ± 6	0.529	0.230	0.846
	LDL, mg/dL	107 ± 11	103 ± 16	107 ± 16	108 ± 17	114 ± 13	118 ± 25	0.965	0.009	0.484
	Triglycerides, mg/dL	115 ± 21	109 ± 19	121 ± 34	140 ± 33	149 ± 39	165 ± 44	0.124	<0.001	0.181
	Glucose, mg/dL	86 ± 8	85 ± 12	88 ± 11	93 ± 12	90 ± 10	95 ± 9	0.138	0.013	0.353
	Insulin, μU/mL	9.14 ± 1.5	10.2 ± 3	9.6 ± 3	12.9 ± 3	9.7 ± 2	14.3 ± 2.5	<0.001	<0.001	0.007
	HOMA-IR	1.94 ± 0.3	2.16 ± 0.7	2.09 ± 0.6	2.98 ± 0.7	2.14 ± 0.4	3.35 ± 0.7	<0.001	<0.001	0.003
	Testosterone, nmol/L	1.55 ± 0.7	2.51 ± 1.1	1.59 ± 0.4	3.81 ± 1.7	1.62 ± 0.9	4.08 ± 1.6	<0.001	0.005	0.011
	Androstenedione, nmol/L	4.22 ± 2.1	7.78 ± 2.9	4.26 ± 1.5	8.92 ± 3.1	4.45 ± 2.3	9.29 ± 3.3	<0.001	0.320	0.501
Adipokines Values										
Leptin, ng/mL		16.5 ± 6.9	18.1 ± 7.9	22.6 ± 7.2	24.7 ± 9.1	33.5 ± 8.3	33.1 ± 9	0.460	<0.001	0.760
Adiponectin, ng/mL		12.3 ± 5.4	11.8 ± 5.3	8.7 ± 5.9	8.7 ± 5.7	7.1 ± 4.8	7.3 ± 4.4	0.885	<0.001	0.955
TNF-α, pg/mL		3.8 ± 2.9	4.2 ± 3.1	4.4 ± 2.4	5.4 ± 1.9	5.7 ± 1.9	6.9 ± 2.6	0.048	<0.001	0.783
IL-6, pg/mL		1.8 ± 1.8	2.0 ± 1.8	2.1 ± 1.5	3.2 ± 2.5	4.2 ± 2.7	6.7 ± 3.4	0.004	<0.001	0.109

Data are presented as mean ± standard deviation. PCOS: polycystic ovary syndrome; mg/dL: milligram per deciliter; HDL: high-density lipoprotein; LDL: low-density lipoprotein; μU/mL,: micro units per milliliter; HOMA-IR: homeostatic model assessment for insulin resistance; nmol/L: nanomole per liter; ng/mL: nanograms per milliliter; TNF-α: tumor necrosis factor alpha; pg/mL: picogram per milliliter; IL-6: interleukin-6.

Table 3 shows the characteristics and hemodynamic parameters, and Table 4 presents the metabolic parameters and adipokines values of the PCOS groups before and after 16 weeks of aerobic physical training. After the 16 weeks of aerobic physical training there were reduction in body fat percentage, HR and systolic BP (SBP). It also increased VO_2peak_. However, the inverse relationship between the body fat percentage and VO_2peak_ remained. In metabolic parameters, after the physical training the high-density lipoprotein (HDL) showed higher values and the triglyceride, insulin and HOMA-IR showed lower values than before training. In turn, the groups with high body fat percentage presented increased total cholesterol, LDL, triglyceride, glucose, insulin, testosterone, and androstenedione levels and HOMA-IR values. For adipokines, after the physical training there were increased adiponectin and reduced leptin and IL-6 levels. Even though, the groups with high body fat percentage still presented low levels of adiponectin and increased leptin, TNF-α, and IL-6 levels.

**Table 3:** Characteristics and hemodynamics parameters obtained before and after aerobic physical training in PCOS groups

		22%-27%		28%-32%		33%-37%		Body Fat Factor	Training Factor	Interaction
		Before	After	Before	After	Before	After	P	P	P
Characteristics										
	Age, years	34 ± 5	-	35 ± 7	-	33 ± 7	-	-	-	-
	Height, cm	162 ± 7	-	165 ± 5	-	161 ± 6	-	-	-	-
	Weight, kg	59 ± 4	58 ± 4	69 ± 6	67 ± 6	76 ± 9	74 ± 8	<0.001	0.150	0.946
	BMI, kg/m^2^	22.6 ± 2.9	22.1 ± 2.9	25.5 ± 3	24.7 ± 2,9	29.9 ± 4.5	28.8 ± 4.4	<0.001	0.314	0.977
	Body fat percentage	24.4 ± 2.1	23.8 ± 2.3	30.3 ± 1.5	28.4 ± 1.5	35.2 ± 1.5	34.4 ± 1.9	<0.001	0.001	0.271
Hemodynamic Parameters										
	HR, bpm	76 ± 10	68 ± 9	78 ± 5	69 ± 8	77 ± 9	68 ± 8	0.811	<0.001	0.994
	SBP, mmHg	115 ± 13	109 ± 12	112 ± 9	107 ± 9	118 ± 8	112 ± 8	0.056	0.032	0.771
	DBP, mmHg	73 ± 13	70 ± 12	71 ± 9	70 ± 9	78 ± 9	74 ± 9	0.063	0.187	0.597
	MBP, mmHg	90 ± 13	85 ± 12	87 ± 9	85 ± 8	94 ± 8	89 ± 8	0.051	0.086	0.681
	VO_2peak_, mL/kg/min	30 ± 3	34 ± 3	29 ± 3	33 ± 3	25 ± 6	29 ± 5	<0.001	<0.001	0.970

Data are presented as the mean ± standard deviation. PCOS: polycystic ovary syndrome; BMI: body mass index; HR: heart rate; bpm: beats per minute; SBP: systolic blood pressure; DBP: diastolic blood pressure; MBP: mean blood pressure; VO_2peak_: oxygen consumption at peak of exercise; mL: milliliter; kg: kilogram; min: minute.

**Table 4:** Metabolic and adipokines parameters obtained before and after 16 weeks of aerobic physical training in PCOS groups

		22%-27%		28%-32%		33%-37%		Body Fat Factor	Training Factor	Interaction
		Before	After	Before	After	Before	After	P	P	P
Metabolic Values										
	Total Cholesterol, mg/dL	200 ± 24	191 ± 23	205 ± 32	198 ± 28	219 ± 32	210 ± 27	0.011	0.098	0.987
	HDL, mg/dL	44 ± 8	47 ± 7	42 ± 5	46 ± 5	41 ± 6	45 ± 6	0.172	0.002	0.895
	LDL, mg/dL	103 ± 16	101 ± 12	108 ± 17	102 ± 14	118 ± 25	109 ± 19	0.009	0.093	0.624
	Triglycerides, mg/dL	109 ± 19	111 ± 15	140 ± 33	118 ± 26	165 ± 44	132 ± 27	<0.001	0.001	0.027
	Glucose, mg/dL	85 ± 12	87 ± 8	93 ± 12	86 ± 6	95 ± 9	92 ± 7	0.004	0.121	0.116
	Insulin, μU/mL	10.2 ± 3	9.8 ± 2.1	12.9 ± 3	10 ± 1.8	14.3 ± 2.5	11 ± 2	<0.001	<0.001	0.018
	HOMA-IR	2.16 ± 0.7	2.11 ± 0.5	2.98 ± 0.7	2.16 ± 0.4	3.35 ± 0.7	2.5 ± 0.6	<0.001	<0.001	0.010
	Testosterone, nmol/L	2.51 ± 1.1	2.56 ± 0.8	3.81 ± 1.7	4.15 ± 1.3	4.08 ± 1.6	4.77 ± 1.5	<0.001	0.164	0.598
	Androstenedione, nmol/L	7.78 ± 2.9	6.92 ± 2.3	8.92 ± 3.1	8.18 ± 2.7	9.29 ± 3.3	9.48 ± 2	0.006	0.356	0.653
Adipokines Values										
	Leptin, ng/mL	18.1 ± 7.9	14.5 ± 6	24.7 ± 9.1	17.9 ± 6.5	33.1 ± 9	24.5 ± 6.5	<0.001	<0.001	0.354
	Adiponectin, ng/mL	11.8 ± 5.3	13.3 ± 5.3	8.7 ± 5.7	11.9 ± 5	7.3 ± 4.4	10.4 ± 3.8	0.005	0.005	0.683
	TNF-α, pg/mL	4.2 ± 3.1	4.0 ± 2.2	5.4 ± 1.9	5.8 ± 1.5	6.9 ± 2.6	6.4 ± 2.1	<0.001	0.862	0.668
	IL-6, pg/mL	2.0 ± 1.8	2.3 ± 1.4	3.2 ± 2.5	2.8 ± 1.9	6.7 ± 3.4	4.3 ± 1.6	<0.001	0.046	0.019

Data are presented as mean ± standard deviation. PCOS: polycystic ovary syndrome; mg/dL: milligram per deciliter; HDL: high-density lipoprotein; LDL: low-density lipoprotein; μU/mL: micro units per milliliter; HOMA-IR: homeostatic model assessment for insulin resistance; nmol/L: nanomole per liter; ng/mL: nanograms per milliliter; TNF-α: tumor necrosis factor alpha; pg/mL: picogram per milliliter; IL-6: interleukin-6.

Table 5 shows the characteristics and hemodynamic parameters, and Table 6 presents the metabolic parameters and adipokine values of the control groups before and after 16 weeks of aerobic physical training. After the physical training, the groups showed lower body fat percentage, HR, and BP values compared with before training. It also increased VO_2peak_. Nevertheless, the inverse relationship between the body fat percentage and VO_2peak_ remained. With regard to metabolic parameters, after the physical training there was increase in HDL levels and reduction in triglyceride levels. In turn, the groups with high body fat percentage showed increased values of total cholesterol, LDL, triglyceride, and HOMA-IR. Table 6 also shows that after physical training there were increase in adiponectin and decrease in leptin and IL-6 levels. However, the groups with high body fat percentage showed reduced adiponectin and increased leptin, TNF-α, and IL-6 levels.

**Table 5:** Characteristics and hemodynamic parameters obtained before and after aerobic physical training in control groups

		22%-27%		28%-32%		33%-37%		Body Fat Factor	Training Factor	Interaction
		Before	After	Before	After	Before	After	P	P	P
Characteristics										
	Age, years	32 ± 7	-	33 ± 7	-	31 ± 4	-	-	-	-
	Height, cm	165 ± 7	-	166 ± 7	-	163 ± 5	-	-	-	-
	Weight, kg	63 ± 6	61 ± 5	72 ± 7	68 ± 6	79 ± 11	77 ± 10	<0.001	0.064	0.727
	BMI, kg/m^2^	23.1 ± 2.6	22.3 ± 2.2	26.3 ± 3	24.7 ± 2.4	29.8 ± 4.3	29.2 ± 3.6	<0.001	0.088	0.767
	Body fat percentage	23.6 ± 1.7	22.9 ± 1.8	29.8 ± 3.2	26.4 ± 2.6	35.7 ± 3.2	34.5 ± 2.3	<0.001	<0.001	0.050
Hemodynamic Parameters										
	HR, bpm	74 ± 13	67 ± 9	73 ± 8	64 ± 6	76 ± 12	69 ± 1	0.268	<0.001	0.893
	SBP, mmHg	111 ± 11	106 ± 22	113 ± 11	107 ± 8	117 ± 10	112 ± 7	0.020	0.004	0.925
	DBP, mmHg	71 ± 7	70 ± 9	76 ± 9	70 ± 8	75 ± 9	74 ± 7	0.115	0.030	0.285
	MBP, mmHg	87 ± 9	84 ± 10	91 ± 10	85 ± 8	92 ± 9	89 ± 7	0.052	0.010	0.557
	VO_2peak_, mL/kg/min	31 ± 3	34 ± 3	28 ± 5	31.5 ± 4	26 ± 5	30.5 ± 5	<0.001	<0.001	0.699

Data are presented as the mean ± standard deviation. BMI: body mass index; HR: heart rate; bpm: beats per minute; SBP: systolic blood pressure; DBP: diastolic blood pressure; MBP: mean blood pressure; VO_2peak_: oxygen consumption at peak of exercise; mL: milliliter; kg: kilogram; min: minute.

**Table 6:** Metabolic and adipokines parameters obtained before and after aerobic physical training in control groups

		22%-27%		28%-32%		33%-37%		Body Fat Factor	Training Factor	Interaction
		Before	After	Before	After	Before	After	P	P	P
Metabolic Values										
	Total Cholesterol, mg/dL	196 ± 17	190 ± 12	212 ± 45	201 ± 30	209 ± 16	206 ± 28	0.041	0.175	0.822
	HDL, mg/dL	45 ± 8	49 ± 7	42 ± 11	47 ± 9	43 ± 7	46 ± 7	0.314	0.014	0.862
	LDL, mg/dL	107 ± 11	110 ± 10	107 ± 16	111 ± 22	114 ± 13	111 ± 11	0.038	0.609	0.502
	Triglycerides, mg/dL	115 ± 21	110 ± 11	121 ± 34	113 ± 21	149 ± 39	123 ± 25	<0.001	0.009	0.175
	Glucose, mg/dL	86 ± 8	85 ± 5	88 ± 11	86 ± 5	90 ± 10	89 ± 7	0.102	0.373	0.959
	Insulin, μU/mL	9.14 ± 1.5	8.54 ± 1.6	9.6 ± 3	8.9 ± 2.1	9.7 ± 2	9.4 ± 2	0.275	0.159	0.904
	HOMA-IR	1.94 ± 0.3	1.79 ± 0.3	2.09 ± 0.6	1.9 ± 0.4	2.14 ± 0.4	2.1 ± 0.4	0.049	0.081	0.831
	Testosterone, nmol/L	1.55 ± 0.7	1.66 ± 0.6	1.59 ± 0.4	1.73 ± 0.4	1.62 ± 0.9	1.72 ± 0.7	0.887	0.322	0.994
	Androstenedione, nmol/L	4.22 ± 2.1	4.15 ± 1.7	4.26 ± 1.5	4.37 ± 1.2	4.45 ± 2.3	4.04 ± 1.2	0.941	0.688	0.790
Adipokines Values										
	Leptin, ng/mL	16.5 ± 6.9	13.5 ± 3.4	22.6 ± 7.2	13.9 ± 3.9	33.5 ± 8.3	18.9 ± 6.7	<0.001	<0.001	<0.001
	Adiponectin, ng/mL	12.3 ± 5.4	14.3 ± 4.5	8.7 ± 5.9	11.1 ± 5.9	7.1 ± 4.8	10.4 ± 3.8	<0.001	0.007	0.840
	TNF-α, pg/mL	3.8 ± 2.9	3.8 ± 1.9	4.4 ± 2.4	4.1 ± 1.7	5.7 ± 1.9	4.9 ± 1.5	0.006	0.424	0.712
	IL-6, pg/mL	1.8 ± 1.8	1.8 ± 1.3	2.1 ± 1.5	1.8 ± 0.9	4.2 ± 2.7	2.6 ± 1.4	<0.001	0.046	0.091

Data are presented as mean ± standard deviation. mg/dL: milligram per deciliter; HDL: high-density lipoprotein; LDL: low-density lipoprotein; μU/mL: micro units per milliliter; HOMA-IR: homeostatic model assessment for insulin resistance; nmol/L: nanomole per liter; ng/mL: nanograms per milliliter; TNF-α: tumor necrosis factor alpha; pg/mL: picogram per milliliter; IL-6: interleukin-6.

Table 7 shows the characteristics and hemodynamic parameters, and Table 8 shows the metabolic parameters and adipokine values of all women with and without PCOS (control groups) distributed according to the body fat percentage. These data were obtained after aerobic physical training. The groups with high body fat percentage showed the highest values of the weight and BMI. It was also showed the lowest VO_2peak_ values and major values of SBP and MBP. Regarding to metabolic parameters, the groups with high body fat percentage showed highest levels of total cholesterol, triglycerides, and glucose. Additionally, they showed minor adiponectin values. Both body fat and PCOS showed high insulin, testosterone, androstenedione, leptin, TNF-α, and IL-6 levels and HOMA-IR values ( Table 8 ).

**Table 7:** Characteristics and hemodynamic parameters of PCOS and control groups, divided according to the body fat percentage, obtained after aerobic physical training

	22%-27%		28%-32%		33%-37%		PCOS Factor	Body Fat Factor	Interaction
	Control (n = 20)	PCOS (n = 20)	Control (n = 20)	PCOS (n = 20)	Control (n = 20)	PCOS (n = 20)	P	P	P
Characteristics									
Age, years	32 ± 7	34 ± 5	33 ± 7	35 ± 7	31 ± 4	33 ± 7	0.084	0.686	0.994
Height, cm	165 ± 7	162 ± 7	166 ± 7	165 ± 5	163 ± 5	161 ± 6	0.109	0.064	0.875
Weight, kg	61 ± 5	58 ± 4	68 ± 6	67 ± 6	77 ± 9	74 ± 8	0.064	<0.001	0.752
BMI, kg/m^2^	22.3 ± 2.2	22.1 ± 2.9	24.7 ± 2	24.7 ± 3	29.2 ± 3.6	28.8 ± 4.4	0.733	<0.001	0.967
Body fat percentage	22.9 ± 1.8	23.7 ± 2.4	26.4 ± 2.6	28.4 ± 1.5	34.5 ± 2.3	34.3 ± 1.9	0.022	<0.001	0.081
Hemodynamic Parameters									
HR, bpm	67 ± 8	68 ± 9	64 ± 5	69 ± 8	69 ± 10	68 ± 8	0.351	0.587	0.346
SBP, mmHg	106 ± 11	109 ± 12	107 ± 8	109 ± 9	112 ± 7	114 ± 7	0.176	0.022	0.959
DBP, mmHg	70 ± 9	70 ± 12	70 ± 8	71 ± 9	74 ± 7	74 ± 9	0.627	0.097	0.867
MBP, mmHg	84 ± 10	85 ± 12	85 ± 8	86 ± 9	89 ± 6	90 ± 7	0.380	0.043	0.967
VO_2peak_, mL/kg/min	34 ± 3	34 ± 3	32 ± 4	33 ± 3	30 ± 5	29 ± 5	0.767	<0.001	0.261

Data are presented as mean ± standard deviation. PCOS: polycystic ovary syndrome; BMI: body mass index; HR: heart rate; bpm: beats per minute; SBP: systolic blood pressure; DBP: diastolic blood pressure; MBP: mean blood pressure; VO_2peak:_ oxygen consumption at peak of exercise; mL: milliliter; kg: kilogram; min: minute.

**Table 8:** Metabolic parameters and adipokines values of PCOS and control groups, divided according to the body fat percentage, obtained after aerobic physical training

		22%-27%		28%-32%		33%-37%		PCOS Factor	Body Fat Factor	Interaction
		Control (n = 20)	PCOS (n = 20)	Control (n = 20)	PCOS (n = 20)	Control (n = 20)	PCOS (n = 20)	P	P	P
Metabolic Values										
	Total Cholesterol, mg/dL	191 ± 12	191 ± 23	201 ± 30	198 ± 28	206 ± 28	210 ± 27	0.940	0.014	0.831
	HDL, mg/dL	49 ± 7	47 ± 7	47 ± 9	46 ± 5	46 ± 7	45 ± 6	0.431	0.268	0.943
	LDL, mg/dL	110 ± 10	101 ± 12	104 ± 10	102 ± 14	111 ± 11	109 ± 19	0.084	0.073	0.381
	Triglycerides, mg/dL	110 ± 11	111 ± 15	113 ± 21	118 ± 26	123 ± 25	132 ± 27	0.220	0.002	0.672
	Glucose, mg/dL	85 ± 5	87 ± 8	86 ± 5	86 ± 6	89 ± 7	92 ± 7	0.204	0.006	0.639
	Insulin, μU/mL	8.5 ± 1.6	9.8 ± 2.1	9.0 ± 2.1	10 ± 1.8	9.4 ± 1.8	11 ± 2	<0.001	0.037	0.825
	HOMA-IR	1.79 ± 0.3	2.11 ± 0.5	1.91 ± 0.4	2.16 ± 0.4	2.07 ± 0.4	2.5 ± 0.6	<0.001	0.003	0.632
	Testosterone, nmol/L	1.66 ± 0.6	2.56 ± 0.8	1.73 ± 0.4	4.15 ± 1.3	1.72 ± 0.7	4.77 ± 1.5	<0.001	<0.001	<0.001
	Androstenedione, nmol/L	4.15 ± 1.7	6.92 ± 2.3	4.37 ± 1.2	8.18 ± 2.7	4.04 ± 1.2	9.48 ± 2	<0.001	0.020	0.010
Adipokines Values										
	Leptin, ng/mL	13.5 ± 3.4	14.5 ± 6	13.9 ± 3.9	17.9 ± 6.5	18.9 ± 6.7	24.5 ± 6.5	<0.001	<0.001	0.178
	Adiponectin, ng/mL	12.3 ± 5.4	13.3 ± 5.3	11.1 ± 5.9	11.9 ± 5	10.4 ± 3.8	10.4 ± 3.8	0.918	0.007	0.695
	TNF-α, pg/mL	3.8 ± 2.9	4.0 ± 2.2	4.1 ± 1.7	5.8 ± 1.5	4.9 ± 1.5	6.4 ± 2.1	0.001	<0.001	0.119
IL-6, pg/mL		1.8 ± 1.8	2.3 ± 1.4	1.8 ± 0.9	2.8 ± 1.9	2.6 ± 1.4	4.3 ± 1.6	<0.001	<0.001	0.256

Data are presented as mean ± standard deviation. PCOS: polycystic ovary syndrome; mg/dL: milligram per deciliter; HDL: high-density lipoprotein; LDL: low-density lipoprotein; μU/mL: micro units per milliliter; HOMA-IR: homeostatic model assessment for insulin resistance; nmol/L: nanomole per liter; ng/mL: nanograms per milliliter; TNF-α: tumor necrosis factor alpha; pg/mL: picogram per milliliter; IL-6: interleukin-6.

## DISCUSSION

Our results indicate that an increase in the body fat percentage is associated with important metabolic and adipokine changes in women with and without PCOS. However, women with PCOS had higher metabolic parameters and adipokine values than women without PCOS. The results also suggest that aerobic physical training is essential, primarily in women with PCOS. It promotes important metabolic adjustments, such as a reduction in triglyceride, blood glucose, insulin, leptin, and IL-6 levels and HOMA-IR values, in addition to increasing adiponectin concentrations. These alterations were particularly salient in the group with the highest percentage of body fat.

In the current study, all PCOS groups presented with laboratory hyperandrogenism. However, only the 28%-32% and 33%-37% groups had increased insulin resistance. The literature suggests that in PCOS, hyperandrogenism and insulin resistance are influenced by positive feedback ( 4 , 11 , 12 , 24 ). It seems that the association of an increased body fat percentage promotes an additive effect in this relationship. In turn, aerobic physical training seems to attenuate this process. We observed significant reductions in insulin concentrations and HOMA-IR values in the 28%-32% and 33%-37% groups, in addition to other positive metabolic effects. This reaffirms the relevance of this non-pharmacological tool in the treatment of women with PCOS ( 2 , 3 , 17 , 18 ).

Our study investigated important adipokines that are frequently altered due to an increase in the body fat percentage. Leptin is a protein that suppresses food intake, increases energy expenditure, and regulates neuroendocrine functions, such as glucose and fat metabolism ( 6 ). However, in overweight and obese individuals, its concentration is increased. This suggests resistance. This condition, together with other inflammatory markers, such as IL-6, was also evaluated in the current study and has been associated with the pathogenesis of atherosclerosis ( 25 , 26 ). Even an isolated increase in this adipokine, regardless of other factors, is considered a risk factor for coronary heart disease ( 27 ). Leptin concentrations appeared to increase with increasing the body fat percentage. Accordingly, the increase in leptin concentration associated with PCOS is very controversial, especially in women with a BMI of less than 25 kg/m^2^ ( 4 , 11 , 28 ). In this case, the consensus is that there is a direct positive relationship between leptin concentrations and an increase in the BMI in these women ( 4 , 11 , 12 ).

On the contrary, after 16-weeks of aerobic physical training, there were significant reductions in leptin concentrations in all groups with higher body fat percentages. The causes of this finding might be related to the potential improvement in leptin sensitivity ( 29 ). There are other reasons that may explain this observation. One of these reasons may correspond to a reduction in weight associated with a reduction in the body fat percentage ( 29 ). Other reasons may involve the reduction of insulin concentrations and HOMA-IR values ( 28 ), although further investigation is warranted.

Adiponectin is an adipokine associated with glycemic regulation, insulin sensitivity, and reduced androgen production by the ovaries ( 4 , 7 , 11 , 30 ). It also has an anti-atherogenic role ( 25 , 31 ). However, its relationship with PCOS is controversial ( 7 , 32 , 33 ). In our study, unlike leptin, adiponectin concentrations were negatively associated with the body fat percentage. However, it is uncertain whether this relationship is totally independent of PCOS ( 10 ). This is primarily because the same result was observed in women without PCOS.

As for the effects of aerobic physical training on adiponectin concentrations, the literature remains incipient ( 33 – 35 ). However, in the current study, aerobic physical training increased adiponectin concentrations, specifically in the groups with a high body fat percentage (28%-32% and 33%-37%). In this study, this increase seemed to be associated with a reduction in the body fat percentage. This result corroborates a previous study that observed a reduction in body weight associated with an increase in adiponectin in obese individuals, with and without PCOS ( 36 ). Another cause could be the reduction in leptin and insulin concentrations ( 13 , 33 , 37 ). In this case, there appears to be an inverse relationship between leptin and adiponectin levels ( 38 ). In addition, physical exercise has been shown to play a favorable role in insulin sensitivity ( 14 , 15 , 18 ). This increase in insulin sensitivity seems to stimulate the increased production of adiponectin ( 39 ).

Regarding concentrations of TNF-α and IL-6, both cytokines are known to negatively influence insulin sensitivity ( 40 , 41 ). They are significant markers of low-grade chronic inflammation in several conditions, such as type 2 diabetes mellitus, obesity, and CVD ( 41 , 42 ). Our results showed that TNF-α and IL-6 levels were higher in women with PCOS than in those without PCOS. These findings corroborate the literature ( 43 , 44 ). Our results suggest that the increased body fat percentage in these women increases the levels of these inflammatory cytokines. However, aerobic physical training did not change TNF-α values, even though it positively interfered with insulin, adiponectin, and leptin levels and HOMA-IR values. These are important regulatory factors of TNF-α concentrations ( 43 , 45 ). In contrast, IL-6 levels were significantly reduced after 16 weeks of aerobic physical training, mainly in the groups with the highest body fat percentage. The cause of this reduction is uncertain. However, it suggests that the reduction of other factors, such as insulin, might have influenced the IL-6 concentration in these women ( 46 ). Nonetheless, further studies are required to identify the mechanisms involved in this response, as aerobic physical training did not affect insulin levels in groups without PCOS.

The study has some limitations, such as the absence of groups with higher percentages of body fat, which could further highlight the negative effects of excess body fat on the regulation of adipokines; the study of women who used contraceptives, evaluating their effects on the investigated parameters; and lack of evaluation of PCOS phenotypes and responses to physical training.

In conclusion, our results showed that a gradual increase in the body fat percentage potentiates metabolic impairment in women with PCOS. The results also confirmed a direct relationship between an increased body fat percentage and changes in adipokine levels. These findings highlight the role of aerobic physical training as an important therapeutic tool in the regulation of adipokines and other metabolic parameters in PCOS. This is especially true in women with a high body fat percentage. On the contrary, aerobic physical training did not seem to affect TNF-α levels. This was observed in all women with and without PCOS.

## References

[1] Fauser BCJM, Tarlatzis BC, Rebar RW, Legro RS, Balen AH, Lobo R (2012). Consensus on women's health aspects of polycystic ovary syndrome (PCOS): the Amsterdam ESHRE/ASRM-Sponsored 3rd PCOS Consensus Workshop Group. Fertil Steril.

[2] Teede HJ, Misso ML, Costello MF, Dokras A, Laven J, Moran L (2018). Recommendations from the international evidence-based guideline for the assessment and management of polycystic ovary syndrome. Fertil Steril.

[3] Wild RA, Carmina E, Diamanti-Kandarakis E, Dokras A, Escobar-Morreale HF, Futterweit W (2010). Assessment of cardiovascular risk and prevention of cardiovascular disease in women with the polycystic ovary syndrome: a consensus statement by the Androgen Excess and Polycystic Ovary Syndrome (AE-PCOS) Society. J Clin Endocrinol Metab.

[4] Delitala AP, Capobianco G, Delitala G, Cherchi PL, Dessole S (2017). Polycystic ovary syndrome, adipose tissue and metabolic syndrome. Arch Gynecol Obstet.

[5] Rostamtabar M, Esmaeilzadeh S, Tourani M, Rahmani A, Baee M, Shirafkan F (2021). Pathophysiological roles of chronic low-grade inflammation mediators in polycystic ovary syndrome. J Cell Physiol.

[6] Matsuzawa Y (2005). White adipose tissue and cardiovascular disease. Best Pract Res Clin Endocrinol Metab.

[7] Orio F, Palomba S, Cascella T, Milan G, Mioni R, Pagano C (2003). Adiponectin levels in women with polycystic ovary syndrome. J Clin Endocrinol Metab.

[8] Hotamisligil GS, Shargill NS, Spiegelman BM (1993). Adipose expression of tumor necrosis factor-alpha: direct role in obesity-linked insulin resistance. Science.

[9] Henry BA, Clarke IJ (2008). Adipose tissue hormones and the regulation of food intake. J Neuroendocrinol.

[10] Cardoso NS, Ribeiro VB, Dutra SGV, Ferriani RA, Gastaldi AC, Araújo JE de (2020). Polycystic ovary syndrome associated with increased adiposity interferes with serum levels of TNF-alpha and IL-6 differently from leptin and adiponectin. Arch Endocrinol Metab.

[11] Glintborg D (2016). Endocrine and metabolic characteristics in polycystic ovary syndrome. Dan Med J.

[12] Spritzer PM, Lecke SB, Satler F, Morsch DM (2015). Adipose tissue dysfunction, adipokines, and low-grade chronic inflammation in polycystic ovary syndrome. Reproduction.

[13] Yoshida H, Ishikawa T, Suto M, Kurosawa H, Hirowatari Y, Ito K (2010). Effects of supervised aerobic exercise training on serum adiponectin and parameters of lipid and glucose metabolism in subjects with moderate dyslipidemia. J Atheroscler Thromb.

[14] Bruce CR, Kriketos AD, Cooney GJ, Hawley JA (2004). Disassociation of muscle triglyceride content and insulin sensitivity after exercise training in patients with Type 2 diabetes. Diabetologia.

[15] Hamdy O, Goodyear LJ, Horton ES (2001). Diet and exercise in type 2 diabetes mellitus. Endocrinol Metab Clin North Am.

[16] Warburton DER, Nicol CW, Bredin SSD (2006). Health benefits of physical activity: the evidence. CMAJ.

[17] Benham JL, Yamamoto JM, Friedenreich CM, Rabi DM, Sigal RJ (2018). Role of exercise training in polycystic ovary syndrome: a systematic review and meta-analysis. Clin Obes.

[18] Harrison CL, Lombard CB, Moran LJ, Teede HJ (2011). Exercise therapy in polycystic ovary syndrome: a systematic review. Hum Reprod Update.

[19] Shele G, Genkil J, Speelman D (2020). A Systematic Review of the Effects of Exercise on Hormones in Women with Polycystic Ovary Syndrome. J Funct Morphol Kinesiol.

[20] Rotterdam ESHRE/ASRM-Sponsored PCOS Consensus Workshop Group (2004). Revised 2003 consensus on diagnostic criteria and long-term health risks related to polycystic ovary syndrome. Fertil Steril.

[21] Jackson AS, Stanforth PR, Gagnon J, Rankinen T, Leon AS, Rao DC (2002). The effect of sex, age and race on estimating percentage body fat from body mass index: The Heritage Family Study. Int J Obes Relat Metab Disord.

[22] Griffin IJ, Cole TJ, Duncan KA, Hollman AS, Donaldson MD (1995). Pelvic ultrasound measurements in normal girls. Acta Paediatr.

[23] Geloneze B, Repetto EM, Geloneze SR, Tambascia MA, Ermetice MN (2006). The threshold value for insulin resistance (HOMA-IR) in an admixtured population IR in the Brazilian Metabolic Syndrome Study. Diabetes Res Clin Pract.

[24] Diamanti-Kandarakis E, Dunaif A (2012). Insulin resistance and the polycystic ovary syndrome revisited: an update on mechanisms and implications. Endocr Rev.

[25] Matarese G, Mantzoros C, La Cava A (2007). Leptin and adipocytokines: bridging the gap between immunity and atherosclerosis. Curr Pharm Des.

[26] Karaduman M, Oktenli C, Musabak U, Sengul A, Yesilova Z, Cingoz F (2006). Leptin, soluble interleukin-6 receptor, C-reactive protein and soluble vascular cell adhesion molecule-1 levels in human coronary atherosclerotic plaque. Clin Exp Immunol.

[27] Wallace AM, McMahon AD, Packard CJ, Kelly A, Shepherd J, Gaw A (2001). Plasma leptin and the risk of cardiovascular disease in the west of Scotland coronary prevention study (WOSCOPS). Circulation.

[28] Zheng SH, Du DF, Li XL (2017). Leptin Levels in Women With Polycystic Ovary Syndrome: A Systematic Review and a Meta-Analysis. Reprod Sci.

[29] Fedewa MV, Hathaway ED, Ward-Ritacco CL, Williams TD, Dobbs WC (2018). The Effect of Chronic Exercise Training on Leptin: A Systematic Review and Meta-Analysis of Randomized Controlled Trials. Sports Med.

[30] Panidis D, Kourtis A, Farmakiotis D, Mouslech T, Rousso D, Koliakos G (2003). Serum adiponectin levels in women with polycystic ovary syndrome. Hum Reprod.

[31] Kubota N, Terauchi Y, Yamauchi T, Kubota T, Moroi M, Matsui J (2002). Disruption of adiponectin causes insulin resistance and neointimal formation. J Biol Chem.

[32] Spranger J, Möhlig M, Wegewitz U, Ristow M, Pfeiffer AFH, Schill T (2004). Adiponectin is independently associated with insulin sensitivity in women with polycystic ovary syndrome. Clin Endocrinol (Oxf).

[33] Al-Eisa E, Gabr SA, Alghadir AH (2017). Effects of supervised aerobic training on the levels of anti-Mullerian hormone and adiposity measures in women with normo-ovulatory and polycystic ovary syndrome. J Pak Med Assoc.

[34] Vu V, Riddell MC, Sweeney G (2007). Circulating adiponectin and adiponectin receptor expression in skeletal muscle: effects of exercise. Diabetes Metab Res Rev.

[35] Aktaş HŞ, Uzun YE, Kutlu O, Pençe HH, Özçelik F, Çil EÖ (2022). The effects of high intensity-interval training on vaspin, adiponectin and leptin levels in women with polycystic ovary syndrome. Arch Physiol Biochem.

[36] Passos MCF, Gonçalves MC (2014). Regulation of insulin sensitivity by adiponectin and its receptors in response to physical exercise. Horm Metab Res.

[37] Weiss EP, Racette SB, Villareal DT, Fontana L, Steger-May K, Schechtman KB (2006). Improvements in glucose tolerance and insulin action induced by increasing energy expenditure or decreasing energy intake: a randomized controlled trial. Am J Clin Nutr.

[38] López-Jaramillo P, Gómez-Arbeláez D, López-López J, López-López C, Martínez-Ortega J, Gómez-Rodríguez A (2014). The role of leptin/adiponectin ratio in metabolic syndrome and diabetes. Horm Mol Biol Clin Investig.

[39] Abbasi F, Chu JW, Lamendola C, McLaughlin T, Hayden J, Reaven GM (2004). Discrimination between obesity and insulin resistance in the relationship with adiponectin. Diabetes.

[40] Borst SE (2004). The role of TNF-alpha in insulin resistance. Endocrine.

[41] Rehman K, Akash MSH, Liaqat A, Kamal S, Qadir MI, Rasul A (2017). Role of Interleukin-6 in Development of Insulin Resistance and Type 2 Diabetes Mellitus. Crit Rev Eukaryot Gene Expr.

[42] Popko K, Gorska E, Stelmaszczyk-Emmel A, Plywaczewski R, Stoklosa A, Gorecka D (2010). Proinflammatory cytokines Il-6 and TNF-α and the development of inflammation in obese subjects. Eur J Med Res.

[43] Gao L, Gu Y, Yin X (2016). High Serum Tumor Necrosis Factor-Alpha Levels in Women with Polycystic Ovary Syndrome: A Meta-Analysis. PLoS One.

[44] Peng Z, Sun Y, Lv X, Zhang H, Liu C, Dai S (2016). Interleukin-6 Levels in Women with Polycystic Ovary Syndrome: A Systematic Review and Meta-Analysis. PLoS One.

[45] Engin A (2017). Adiponectin-Resistance in Obesity. Adv Exp Med Biol.

[46] Xu X, Du C, Zheng Q, Peng L, Sun Y (2014). Effect of metformin on serum interleukin-6 levels in polycystic ovary syndrome: a systematic review. BMC Womens Health.

